# Rapid Recombination Mapping for High-Throughput Genetic Screens in *Drosophila*

**DOI:** 10.1534/g3.113.008615

**Published:** 2013-10-29

**Authors:** Anne L. Sapiro, Robert J. Ihry, Derek L. Buhr, Kevin M. Konieczko, Sarah M. Ives, Anna K. Engstrom, Nicholas P. Wleklinski, Kristin J. Kopish, Arash Bashirullah

**Affiliations:** * Division of Pharmaceutical Sciences, University of Wisconsin-Madison, Madison, Wisconsin 53705-2222; † College of Letters and Sciences or College of Agriculture and Life Sciences, University of Wisconsin-Madison, Madison, Wisconsin 53705-2222; ‡ Cellular and Molecular Biology Graduate Program, University of Wisconsin-Madison, Madison, Wisconsin 53705-2222

**Keywords:** dominant markers, EMS mutagenesis, persistent salivary glands (PSG)

## Abstract

Mutagenesis screens are a staple of classical genetics. Chemical-induced mutations, however, are often difficult and time-consuming to identify. Here, we report that recombination analysis with pairs of dominant visible markers provides a rapid and reliable strategy to map mutations in *Drosophila melanogaster*. This method requires only two generations and a total of six crosses in vials to estimate the genetic map position of the responsible lesion with high accuracy. This genetic map position can then be reliably used to identify the mutated gene through complementation testing with an average of nine deficiencies and Sanger sequencing. We have used this approach to successfully map a collection of mutations from an ethyl methanesulfonate−based mutagenesis screen on the third chromosome. We propose that this method also may be used in conjunction with whole-genome sequencing, particularly when multiple independent alleles of the mutated locus are not available. By facilitating the rapid identification of mutated genes, our mapping strategy removes a primary obstacle to the widespread use of powerful chemical mutagenesis screens to understand fundamental biological phenomena.

Open-ended forward genetic screens remain one of the most powerful genetic approaches to identify critical, and often unexpected, regulators of biological processes. These mutagenesis screens, however, have fallen out of favor because identifying the mutated genes can be a slow and tedious process. The available methods for identifying chemical- and radiation-induced mutations fall into two broad categories, those that rely on recombination analysis and those that do not. Direct mapping methods that do not use recombination mapping are often labor-intensive and/or expensive, making these approaches suitable only for mapping a small number of mutations of high interest. For example, crossing a mutation to all deficiencies that span a chromosome often works, but with the large number of deficiencies required to cover the chromosome, simultaneously mapping many mutations becomes impractical. In theory, whole-genome sequencing (WGS) provides an ideal method for mapping mutations; in practice, however, given that a stock identified in an ethyl methanesulfonate (EMS) mutagenesis screen may reveal hundreds of coding-changing lesions, WGS approaches may require multiple alleles to unambiguously identify the lesion of interest (Blumenstiel *et al.* 2009; Osterloh *et al.* 2012).

Mapping methods that rely on recombination analysis offer a reliable way to map mutations and have been routinely used, essentially unchanged, since Sturtevant proposed the method 100 years ago (Sturtevant 1913). In these methods, the frequency of chromosomal exchange with respect to a reference locus provides an estimated distance along the chromosome. The resolution of recombination-based mapping approaches, however, is determined by the density of markers used and the number of recombinants generated and examined. High density can be achieved with molecular markers such as single-nucleotide polymorphisms (SNPs). The use of SNPs for recombination mapping can identify locations within 50 kb but require hundreds of SNP-detection reactions involving polymerase chain reaction and sequencing on a large number of recombinants, making it expensive and labor-intensive (Martin *et al.* 2001; Chen *et al.* 2008). Similarly, the use of molecularly defined *P*-elements for recombination mapping also can provide high resolution, but this degree of resolution requires scoring approximately 10,000 F2 progeny (Zhai *et al.* 2003). Thus, neither of these recombination-based methods is easily scalable for mapping large collections of mutations generated in forward genetic screens. Moreover, standard mapping strategies using recessive markers, although scalable, cannot achieve comparable resolution and often are equally labor-intensive.

Here, we describe the use of pairs of dominant visible markers as a way to rapidly perform recombination analysis. Compared with mapping with recessive markers, the use of dominant markers offers the ability to reduce the number of generations and crosses required to estimate genetic map positions. Although the use of dominant markers for recombination analysis dates back to the days of the first “fly room” (Bridges and Morgan 1923), the use of these markers for mapping was likely abandoned because good dominant markers are rare and thus cannot provide sufficient density for accurate mapping. However, in our strategy, the goal of simultaneously using pairs of dominant markers is not to identify the true genetic map position of a mutation, but instead to rapidly estimate its position relative to the two markers and use this information to guide subsequent steps with either WGS or molecularly-defined deficiencies. We demonstrate the usefulness of using pairs of dominant markers through the mapping of a collection of mutations identified in an EMS-based mutagenesis screen. We had previously identified 38 complementation groups on the third chromosome that selectively disrupt the ecdysone-triggered destruction of larval salivary glands, resulting in a persistent salivary gland (PSG) phenotype (Wang *et al.* 2008). Thirty of these complementation groups were represented by single alleles. Employing pairs of dominant markers, we estimated the genetic map positions of recessive lethal mutations in two generations and six crosses. The positional information obtained is of sufficiently high resolution to map the mutated gene through complementation tests with less than 10 deficiencies. Thus, the use of pairs of dominant markers overcomes existing barriers to the identification of genes through forward genetic approaches by providing a fast, simple, and affordable alternative for recombination mapping.

## Materials and Methods

### Fly stocks and husbandry

The following stocks with dominant markers were obtained from the Bloomington Drosophila Stock Center (BDSC): R,D (FBst0001689), Gl,Sb (FBst0000510), Sb,H (FBst0000586), and H,Pr (FBst0000516). Also from BDSC but not listed are the BSC (Cook *et al.* 2012), DrosDel (Ryder *et al.* 2007), and Exilixis (Parks *et al.* 2004) stocks carrying molecularly defined deletions, included in the deficiency kit on the third chromosome (DK3), that were used for complementation tests. The mutations with a PSG phenotype were identified in a large-scale EMS-mutagenesis screen on the third chromosome (Wang *et al.* 2008). All flies were raised on cornmeal molasses media with yeast, and crosses were performed at room temperature with three to six virgins and at least two males per vial.

### Scoring dominant markers

**Figure 1:**
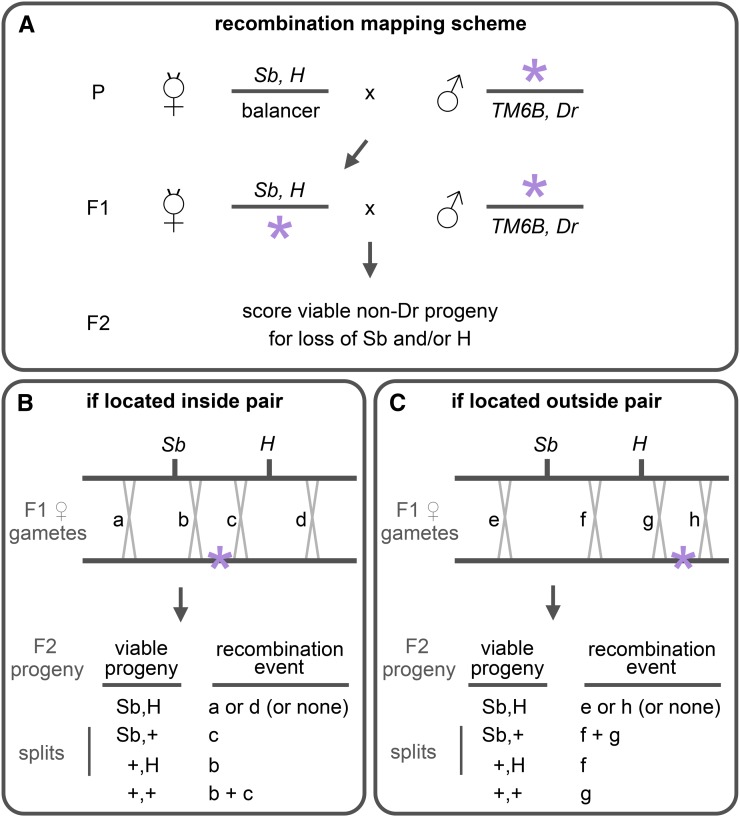
Using pairs of dominant markers for recombination analysis. The figure illustrates the concept of recombination analysis with pairs of dominant markers, using the Sb,H pair as an example. (A) In the parental cross (P), mutant animals are crossed to a stock that contains two dominant markers on the same chromosome, and the resulting female progeny are selected for presence of these markers and the chromosome carrying the mutation of interest (*i.e.*, no balancer; in this case, no Dr progeny). In the F1 cross, these females are then backcrossed to males from the mutant stock, and the viable nonbalancer F2 progeny are scored for the loss of Sb and/or H (*i.e.*, only score Sb,+, +,H and +,+ progeny). (B-C) The panels illustrate the possible recombination events between the marked and mutant chromosomes depending on whether the mutation (indicated with a purple asterisk) is located inside (B) or outside (C) of the two pairs of markers. (B) If a recombination event occurs in “a” or “d” (outside the pair), the recombinant chromosome will be viable, resulting in Sb,H progeny. If recombination occurs in “b,” the recombinant chromosome containing only Sb will be lethal while the recombinant progeny containing only H will be viable, resulting in +,H progeny. Conversely, if the recombination occurs in “c,” the recombinant chromosome containing Sb will be viable whereas the recombinant progeny containing H will now be lethal, resulting in Sb,+ progeny. The ratio of these recombination “splits” between markers reflects the relative position of the mutation. In addition, an important consequence of the mutation being inside is that viable progeny that have lost both markers (+,+ progeny) will only appear if a rare double recombination event occurs in “b” and “c.” (C) As with the preceding example, recombination events in “e” and “h” will result in viable Sb,H progeny. However, recombination events in “f” will result in only +,H viable progeny (Sb,+ progeny would only be viable in the case of a double recombination event in “f” and “g”). Importantly, the distinguishing characteristic of mutations located outside the pair of markers, unlike those located inside, is that unmarked progeny (+,+) can be generated by a single recombination event (in “g”). Thus, the number of viable recombinant progeny that have lost one or both markers is used in the following manner: (1) if the unmarked progeny is absent or rare, then the mutation is inside the markers and the ratio of the “splits” estimates the relative position of the mutation within the pair; and (2) if the unmarked progeny is common, then the mutation is outside the markers and the ratio of the “splits” indicates direction of the mutation. In the example shown, Sb,+ would be less frequent than +,H indicating that the mutation is to the right of the pair. Given that viable Sb,H progeny can also result in the absence of recombination, we do not score this class for mapping.

**Figure 2:**
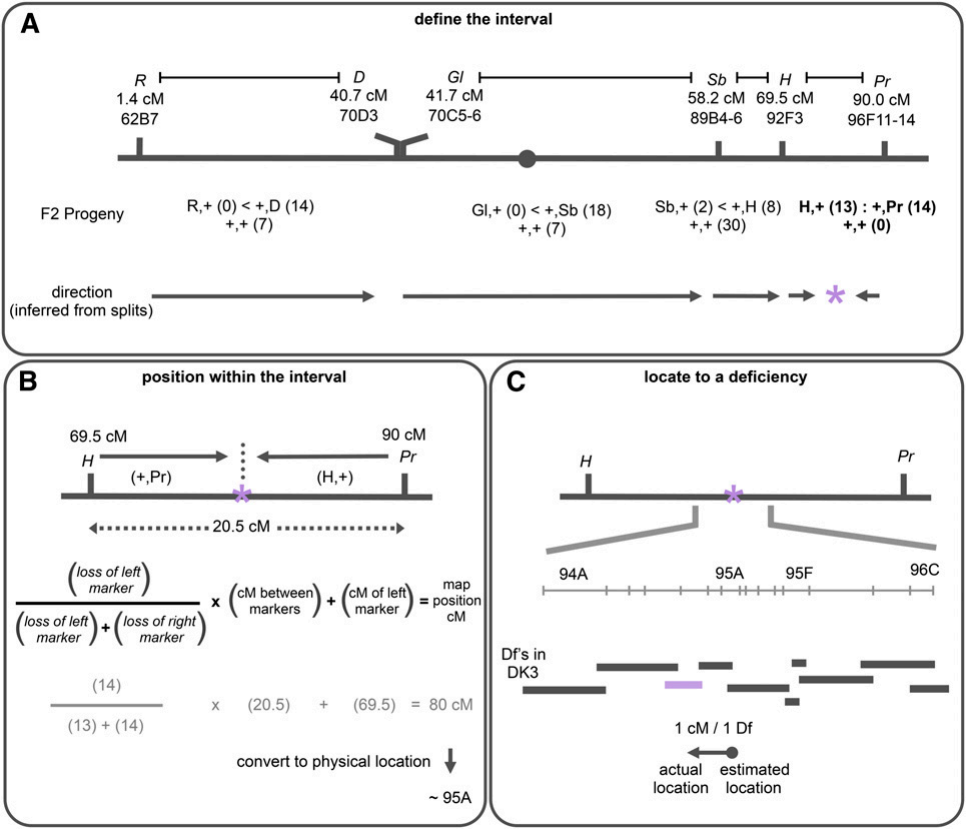
Use of pairs of dominant markers to map a lethal mutation on the third chromosome. An example of the mapping process illustrating the effectiveness of using pairs of dominant markers. (A) The analysis described in Figure 1 is conducted with four pairs of dominant markers (R,D, Gl,Sb, Sb,H, and H,Pr) to map a lethal mutation on the third chromosome, *psg24* (indicated with a purple asterisk). Scoring the viable F2 progeny indicates that only one pair has no unmarked progeny, thus the mutation is located inside the H,Pr pair. Consistent with this interpretation, the ratio of “splits” in the R,D, Gl,Sb and Sb,H crosses point to the H,Pr region. (B) The recombinant “splits” in H,Pr are used to calculate the approximate location of *psg24*. The formula provides the relative distance of the mutation from the left marker. This distance is indicated by the frequency of loss of the left marker among the viable F2 progeny “splits.” In this case, the estimated genetic map position for *psg24* is approximately 80 cM. This genetic location is then used to estimate a cytological location with positional information of known genes (see File S1), estimating the physical location of *psg24* to around 95A. (C) We used complementation tests with deficiencies near 95A to identify the actual location of *psg24*. The mutation was crossed to 10 deficiencies from the DK3 collection spanning the region from 94A to 96C, and it failed to complement Df(3R)BSC619 in 94E, which is about 1 cM or approximately one deficiency away from the initial estimated physical map position. The arrow reflects the approximate reliability of the recombination analysis, where the base of the arrow (dot) represents the estimated genetic map position and the arrowhead represents the actual physical location.

Recombinant F2 progeny were scored for presence of the mapping dominant markers and for absence of the balancer dominant marker (see genetic scheme in Figure 1A). Phenotypes of dominant markers used for mapping are as follows: Roughened (R), scored for rough eye; Dichaete (D), scored for extended wings and missing alulae; Glued (Gl), scored for smaller rough eyes; Stubble (Sb), scored for short and thick bristles on the notum; Hairless (H), scored for loss of the postvertical bristles in the head; and Prickly (Pr), scored for short and thin-tipped bristles on the notum (of note, H and Pr together cause a loss of bristles). For the purpose of this mapping method, the relevant F2 progeny are those that have lost one or both markers; for example, when the Sb,H pair for mapping is used, the relevant progeny to be scored are Sb,+, +,H and +,+ (the remaining progeny are not needed to calculate relative position; see Figure 2B). The balancer used was marked by Drop (TM6B,Dr), which shows a near-complete ablation of the eyes. The use of the Dr-containing balancer facilitates sorting of F2 progeny (the Dr eye phenotype is considerably stronger than those of R and Gl and thus easily identified even in their presence). Although we describe this recombination mapping method for the third chromosome, the principle of using pairs of dominant markers can be applied for mapping mutations on other chromosomes. The second chromosome has a number of good dominant markers that can be used for recombination mapping (see Supporting Information, Table S1 for examples). Moreover, even though dominant visible mutations are rare on the X chromosome, the principle of pairs of dominant markers can be applied by scoring recessive markers in male recombinant progeny.

### Converting genetic map positions into physical map positions

Conversions between genetic and physical map positions, given that they do not have a linear relationship along the chromosome, are performed individually by comparing the positional information of nearby genes. The available genome sequence of *Drosophila melanogaster* is not cross-referenced with genetic map positions. In fact, only a limited percentage of genes have experimentally derived genetic map positions. An extensive list of genes with genetic map positions and their corresponding physical positions (described by chromosomal cytology) is available in the cytogenetic map generated by Michael Ashburner for Lindsley & Zimm’s now classic “Redbook” (Lindsley and Zimm 1992). This information is also included, when available, on FlyBase Gene Reports (Marygold *et al.* 2013). To facilitate the task of conversion, we added 42 reference loci with experimentally derived genetic map positions to a physical map of the third chromosome that includes deficiencies available in BDSC (File S1). Once a genetic map position is obtained by recombination analysis, we use File S1 to estimate the corresponding physical location. For this study, the deficiencies near this initial estimated physical location were then tested for complementation to validate the recombination mapping analysis.

## Results and Discussion

To test whether recombination analysis with pairs of dominant visible markers provides a fast and efficient method for obtaining genetic map positions, we used this strategy to map newly generated EMS-induced lethal mutations on the third chromosome. The use of dominant markers allows scoring recombinant progeny in the F2 generation, a generation earlier than mapping schemes using recessive markers, eliminating the need to set up crosses with each recombinant progeny. In theory, the use of two broadly spaced markers increases the frequency of recombination events which, in turn, potentially generates positional information with a smaller number of progeny. Although this strategy may reduce the resolution and accuracy of recombination analysis, it allows for rapidly estimating the relative position of a mutation between the two markers. The method is best illustrated by following one class of viable F2 recombinant progeny from a mapping cross: those that have lost both dominant markers present on the mapping chromosome (follow the “+,+” gametes in Figure 1, B and C). When crosses are performed on a small scale (*i.e.*, in vials), the absence or presence of very few unmarked recombinant progeny (carrying the “+,+” gamete) indicates that the mutation is located inside that pair of markers. Given that three to four pairs of dominant markers can span the entire chromosome, only one of these pairs will show that the mutation is inside its boundaries. In this latter cross, the ratio of recombinant progeny that have lost only one dominant marker (see “splits” in Figure 1B) provides the relative position of the mutation within these markers. In the remaining crosses, the ratio of these “splits” indicates direction, whether the mutation is to the left or the right of its boundaries (Figure 1C).

We used four pairs of dominant markers to span the third chromosome: R,D on the left arm, Gl,Sb across the centromere, and Sb,H and H,Pr on the right arm. These pairs of markers are approximately 20 cM apart, except for R and D, which are approximately 40 cM apart. Heterozygous females undergoing recombination—between a pair of markers and the mutation of interest—were backcrossed to males carrying the mutation and a TM6B,Dr balancer (Figure 1A). The progeny carrying recombinant female gametes that have lost the mutation (viable nonbalancer progeny) were then scored for the loss of one or both dominant markers used. In practice, scoring is very fast: Dr progeny are easily identified and moved aside, and then the progeny that have lost one or both of the two markers are scored (scoring progeny with both markers is not informative; see Figure 1). Figure 2 illustrates the mapping results for a lethal mutation generated in our EMS screen, *psg24*. Only the H,Pr cross lacks unmarked recombinant progeny (Figure 2A), indicating that the mutation is located inside its boundaries. Consistently, the ratio of “splits” in the other crosses points to the mutation being in H,Pr (*e.g.*, more +,D than R,+ progeny indicates the mutation is to the right of D; Figure 2A). Then, the ratio of “splits” between H and Pr was used to calculate the relative position of the mutation within the pair of markers (Figure 2B). Thus, we estimate that the mutation lies halfway between H and Pr (a 13:14 ratio) at approximately 80 cM (Figure 2B).

To demonstrate that the estimated genetic map position could be used for gene identification, we used deletion mapping with the BDSC deficiency kit for the third chromosome (DK3). Given that these deficiencies are not associated with genetic locations, we converted the estimated genetic map position of our mutation (in cM) to a physical map position (in cytology) (see File S1). This conversion estimated the *psg24* mutation to roughly 95A (Figure 2B). We then tested 10 nearby deficiencies from the deficiency kit for complementation—the mutation failed to complement one of these, Df(3R)BSC619 in cytological band 94E (Figure 2C). Thus, the estimated genetic location for *psg24* was approximately 1 cM away from the deficiency that uncovered it (genetic map position of the deficiency was estimated using available genetic map positions of genes contained within it). Another way to measure the distance between the estimated genetic map position and the actual physical map position is to use the number of DK3 deficiencies that separate them; in this case, the estimated and actual positions are one deficiency apart. The latter is a more practical measure of the work required to locate a mutation after recombination mapping. Importantly, these results demonstrate that, even when scoring a relatively small number of F2 recombinant progeny, recombination mapping with pairs of dominant markers works.

To determine whether this method could be used to facilitate mapping after large-scale forward genetic screens, we used pairs of dominant markers to simultaneously map a collection of mutations identified in an EMS-mutagenesis screen on the third chromosome. The mapping process described above for *psg24* was simultaneously applied to 38 complementation groups of recessive lethal mutations that had a PSG phenotype (Table S2). As a result, we successfully mapped 30 of the 38 complementation groups to deficiencies within the DK3 (Table 1). All mutations within the 30 complementation groups failed to complement at least one other deficiency within their respective region, further validating the location of the lethal mutations. Moreover, subsequent analysis showed that the PSG phenotypes also map to the same locations, validating the ability to map the loci of interest. In two separate cases, complementing mutations mapped to the same gene, reducing the number of mapped loci to 28 (Table 1). Among the mutations we failed to map, one maps to a region of the third chromosome without available deficiencies and was not pursued. Given that approximately 98% of the third chromosome is covered by deficiencies (Cook *et al.* 2012), this is expected to be a rare occurrence. The remaining seven unmapped mutations had inconsistent mapping results that were likely caused by multiple independent and/or interacting lesions on the same chromosome—a common occurrence with alleles generated in chemical mutagenesis screens. Thus, our results demonstrate that we were able to map the great majority (79%) of the mutations identified in the EMS screen; these mapped mutations likely include most, if not all, of the loci with a tractable PSG phenotype. Taken together, these results demonstrate that using pairs of dominant markers is effective and can be easily scaled to map a large collection of chemically‐induced lethal mutations.

**Table 1:** Mapping the collection of PSG mutations using pairs of dominant markers

		Estimated		Actual	
mutation	Alleles	cM	cyto	Df	cyto
*psg8*	2	left of *R*	61A	Df(3L)BSC362	61C
*psg28*	1	left of *R*	61A	Df(3L)BSC362	61C
*psg2**^a^*	2	25	66CD	Df(3L)Exel6105	64D
*psg5**^a^*	2	34	67EF	Df(3L)Exel6112	66B
*psg15*	1	28.1	67B	Df(3L)BSC388	66B
*psg23*	1	23.5	66C	Df(3L)BSC673	67BD
*psg16*	1	40.7	70C	Df(3L)ED4457	68A
*psg19*	1	48.3	85AB	Df(3L)ED217	71B
*psg3**^a^*	2	44.4	72D	Df(3L)BSC774	72D
*psg27*	1	43.8	72A	Df(3L)ED4606	72D
*psg21*	1	43.8	72A	Df(3L)BSC775	75E
*psg22*	1	52.6	88B	Df(3L)BSC775	75E
*psg10*	1	55	88F	Df(3L)ED229	76A
*psg14*	1	45.6	73F	Df(3L)ED229	76D
*psg26*	1	50.5	87A	Df(3L)ED5100	82D
*psg4*	3	48.7	85D	Df(3R)Tpl10	83E
*psg9**^b^*	1	44.2	72C	Df(3R)BSC466	85A
*psg20*	1	44.8	72F	Df(3R)BSC507	85D
*psg6*	2	50.3	86F	Df(3R)BSC486	87D
*psg7**^a^*	3	66	91F	Df(3R)Exel6178	90F
*psg25*	1	69.5	92F	Df(3R)ED5938	92A
*psg29*	1	63.8	91C	Df(3R)BSC677	93D
*psg11*	1	70.3	93B	Df(3R)BSC677	93F
*psg24*	1	80.1	95A	Df(3R)BSC619	94E
*psg13*	1	79.8	95A	Df(3R)ED6187	95F
*psg18*	1	86.8	96C	Df(3R)Exel6203	96E
*psg17*	1	right of *Pr*	99A	Df(3R)BSC501	99A
*psg12*	1	right of *Pr*	100D	Df(3R)BSC503	99F

Each of the 28 complementation groups, and the number of alleles in each group, are indicated in the first and second columns, respectively. The third column displays the genetic map location obtained by using the pairs of dominant markers for recombination analysis; the fourth column displays the corresponding estimated cytological location. The fifth and sixth columns represent the deficiencies that fail to complement each complementation group and their cytological location, respectively. The first two mutants (*psg8* and *psg28)* map to the left of *R*, and the last two mutant (*psg17* and *psg12)* map to the right of *Pr*. The location of these four mutations was based on the orientation of splits because they were not inside any of the pairs used; however, given that these regions are covered by a small number of DK3 stocks, deficiency mapping was straight forward. PSG, persistent salivary gland.

a Recombination results reported by Wang *et al.* 2008.

b Recombination results reported in Ihry *et al.* 2012.

**Figure 3:**
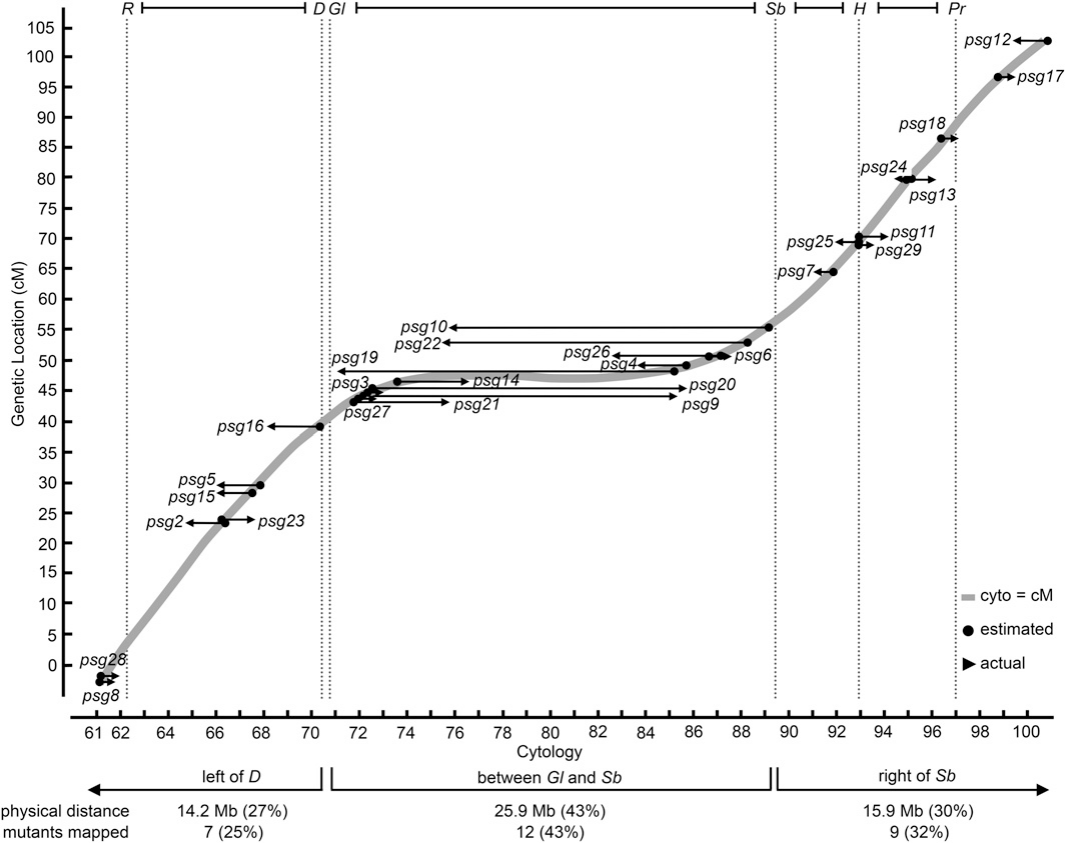
Reliability of using pairs of dominant markers for mapping a collection of EMS-induced lethal mutations. The graph illustrates the relationship between genetic and cytological map positions across the third chromosome (thick gray line). The estimated genetic locations of the 28 mapped mutations are shown by the dots at the base of arrows on the gray line, with arrowheads pointing to actual cytological location as determined by complementation tests with deficiencies. Thus, the length of the resulting arrows reflects the reliability of the mapping process. Not surprisingly, the regions of greater reliability (reflected by shorter arrows) correlate with the regions that have a linear relationship between the genetic and physical map positions (*i.e.*, left of *D* and right of *Sb*). The number of mutations that map to each region is proportional to the physical size of these regions, indicating the ability to map mutations was not affected by their chromosomal location.

To evaluate the overall reliability of using pairs of dominant markers for recombination analysis, we determined the distance between the estimated and actual map positions for all mapped mutations. Each mutation is represented by an arrow where the base (marked by a dot) indicates the estimated genetic map positions, and the arrowhead indicates the actual physical map positions (Figure 2C and Figure 3). Thus, the length of the arrow reflects the relative reliability of the recombination data generated by this method. The “arrows” for all 28 mapped complementation groups are displayed within a graph that illustrates the average relationship between genetic and physical map positions on the third chromosome (gray line, modified from Ashburner 1989). The most striking feature of this relationship is the nonlinearity along the chromosome caused by the inhibitory effects of the centromere on recombination rates. Not surprisingly, the reliability of our recombination mapping parallels this relationship and reflects the inefficiency of recombination-based methods to map mutations located between Gl and Sb due to the centromere effect (Figure 3). This region represents 43% of the third chromosome based on nucleotides and contains the same fraction of the mutations generated in our screen (Figure 3). We found that mutations located between *Gl* and *Sb* are best mapped by crossing them to all DK3 deficiencies that cover this region. On the other hand, mutations located within the remaining ~60% of the chromosome were easily and reliably mapped to deficiencies. This is illustrated by the relatively short “arrows” separating the estimated genetic and actual physical map positions (Figure 3). Moreover, by measuring this distance in terms of DK3 deficiencies, mutations located on the left arm are mapped by crossing them to, on average, 10 DK3 deficiencies (five on either side of the estimated genetic location); mutations located on the right arm require, on average, 8 DK3 deficiencies (four on either side of the estimated genetic location) (calculated from data in Table S2). The difference in resolution between the arms likely reflects the larger distance between the dominant markers on the left arm.

Accurate measurements of genetic map positions is a persnickety endeavor, requiring rigorous control of diverse parameters like temperature and crowding of cultures, age of females, and genetic backgrounds. Moreover, a genetic map position reflects the total frequency of exchange between a locus of interest and a reference gene less than 10 cM away (to minimize interference due to double crossover events) and thus requires scoring all viable progeny. In contrast, our results demonstrate that, by using pairs of dominant markers, we can simplify the process of recombination analysis for the purpose of mapping. Although we attempted to maintain uniform culture conditions for our crosses, we were not fastidious about it. Moreover, we only scored a small subset of the viable recombinant progeny and used markers that were 20 and even 40 cM apart. Despite these simplifications, the empirical evidence presented here indicates that the process is still effective and reliable. In fact, our mapping data indicates that we can simplify the process even further: we can skip the Gl,Sb pair for mapping. Instead of using the Gl,Sb pair, the direction of “splits” in the other three pairs reliably locate mutations that map between Gl and Sb (see Table S3). Thus, recombination analysis can be achieved with three pairs of dominant markers in six crosses total. The overall effectiveness of recombination analysis using pairs of markers is likely twofold. On the one hand, currently available reagents, like the collections of molecularly-defined deficiencies that span the genome (Parks *et al.* 2004; Ryder *et al.* 2007; Cook *et al.* 2012), help reduce the resolution required to translate a genetic map position into a physical location. On the other hand, by using pairs of markers, we are estimating a relative position between the two markers (*e.g.*, 30% of the distance between *H* and *Pr* from *H*), not measuring a true genetic map position (*e.g.*, 6.2 cM to the right of *H*), likely minimizing the effects of environmental and genetic conditions on the ability to estimate genetic map positions.

Taken together, our results demonstrate that using pairs of dominant markers provides a rapid and effective method to identify the genetic map positions of chemically-induced mutations. Requiring only two generations and six crosses in vials, this method is faster and less labor-intensive than other available methods for recombination analysis. We have shown that this method is not only effective in mapping one mutation at a time, it is also easily scalable to simultaneously tackle a large collection of mutations identified from a forward genetic screen. The resulting genetic map positions are reliable and offer high accuracy to guide complementation tests with deficiencies. This method can also be used iteratively to map even larger collections of mutations, effectively replacing the need for complementation tests as the first step after a mutagenesis screen. For example, we successfully mapped the majority of loci in an independent collection of 134 EMS-induced lethal mutations, by first using the R,D pair and then the H,Pr pair. In addition, although we describe this method for mapping lethal mutations, the strategy can be easily adapted to map visible phenotypes. In fact, the concept of using broadly-spaced pairs of markers can be used with molecular markers like SNPs or with any visible markers like GFP, white, or yellow on *P*-elements. Finally, in addition to its practical value in research, we have found this method to be a good didactic tool in our laboratory to introduce new graduate and undergraduate students to genetics—the excitement of mapping your first mutation is undeniable and provides a reliable way to hook, especially undergraduates, to the joys of science.

## Supplementary Material

Supporting Information
