# Supplementary material for: Rapid Recombination Mapping for High-Throughput Genetic Screens in Drosophila
Source: G3 (Bethesda). 2013 Oct 29;3(12):2313–9. doi: 10.1534/g3.113.008615 (PMC3852393; doi:10.1534/g3.113.008615)
Supplement: Supporting Information [file supp_3_12_2313__index.html]

Rapid Recombination Mapping for High-Throughput Genetic Screens in Drosophila — Supporting Information 

# Rapid Recombination Mapping for High-Throughput Genetic Screens in *Drosophila*

## Supporting Information for Sapiro *et al.*, 2013

**Files in this Data Supplement:**

- Supporting Information - File S1 and Tables S1-S3 (PDF, 560 KB)
- File S1 - Modified map of the third chromosome showing BDSC deficiencies and the genetic map positions of reference genes. (PDF, 3 MB)
- Table S1 - Useful dominant markers for mapping on the second chromosome. (PDF, 310 KB)
- Table S2 - Viable F2 recombinant progeny data generated by recombination analysis using pairs of dominant markers. (PDF, 325 KB)
- Table S3 - Reliable mutant directionality even when *Gl,Sb* marker pair is not used. (PDF, 527 KB)
