# Supplementary material for: Rapid Recombination Mapping for High-Throughput Genetic Screens in Drosophila
Source: G3 (Bethesda). 2013 Oct 29;3(12):2313–9. doi: 10.1534/g3.113.008615 (PMC3852393; doi:10.1534/g3.113.008615)
Supplement: Supporting Information [file supp_g3.113.008615_008615SI.pdf]

G3 · Genes | Genomes | Genetics

Rapid recombination mapping for high-throughput genetic screens in *Drosophila*

Anne L. Sapiro[*†‡], Robert J. Ihry[*§‡], Derek L. Buhr[*†], Kevin M. Konieczko[*†], Sarah M. Ives[*†], Anna K. Engstrom[*†], Nicholas P. Wleklinski[*†], Kristin J. Kopish[*†6], Arash Bashirullah[*§1]

[*] Division of Pharmaceutical Sciences, University of Wisconsin-Madison, Madison, WI
[†] College of Letters and Sciences or College of Agriculture and Life Sciences, University of Wisconsin-Madison, Madison, WI
[§] Cellular and Molecular Biology Graduate Program, University of Wisconsin-Madison, Madison, WI
[‡] A.L.S. & R.J.I. contributed equally to this work

[1] Corresponding author: University of Wisconsin-Madison, Division of Pharmaceutical Sciences, Madison, WI 53705. Email: bashirullah@wisc.edu

DOI: 10.1534/g3.113.008615

File S1

Modified map of the third chromosome showing BDSC deficiencies and the genetic map positions of reference genes.

File S1 is available for download at http://www.g3journal.org/lookup/suppl/doi:10.1534/g3.113.008615/-/DC1.

Table S1 Useful dominant markers for mapping on the second chromosome

genotype	name	cM	cytology
S[1]	*Star, Asteroid*	2-1.3	21E4
wg[Sp-1]	*Sternopleural*	2-22	27F1
J[1]	*Jammed*	2-41	31E
amos[Tft]	*Tufted*	2-53.6	36F6
Bl[1]	*Bristle*	2-54.8	38B5
L[rm] or L[2]	*Lobe*	2-72	51A4
nw[D] or nw[B]	*narrow*	2-79.6	54A1
Bc[1]	*Black cells*	2-80.6	54F6
Pu[2]	*Punch*	2-97	57C7
Frd[1]	*Freckled*	2-102.4	59C1-4
Pin[1]	*Pin*	2-107.3	60C6-D1

Most available as stocks in various multiply-marked combinations from BDSC

Table S2 Viable F2 recombinant progeny data generated by recombination analysis using pairs of dominant markers.

		Recombination mapping F2 progeny															
mutation	alleles	*R,D*			*Gl,Sb*			*Sb,H*			*H,Pr*			total scored	cM	cM away	Dfs away
		R,+	*+,D*	*+,+*	*Gl,+*	*+,Sb*	*+,+*	*Sb,+*	*+,H*	*+,+*	*H,+*	*+,Pr*	*+,+*				
psg8	2	3	1	4	9	4	27				11	0	15	74	0	0	2
psg28	1	25	3	7	4	1	31	21	12	83	13	4	51	255	0	0	2
psg2[a]	2	28	42	1										71	15	10	5
psg5[a]	2	20	91	1										112	25	9	9
psg15	1	9	19	0	7	2	19				15	0	15	86	25.2	2.9	6
psg23	1	7	9	0	7	0	17	2	0	17	10	3	25	97	28	4.5	4
psg16	1	0	26	0	3	1	0				2	0	4	36	35	5.7	6
psg19	1	0	11	2	10	7	2	13	4	59	27	0	26	161	43.6	4.7	39
psg3	2	0	124	9	14	3	0							150	43.6	0.8	1
psg27	1	0	5	1	6	1	0	14	1	6	14	3	13	64	43.6	0.2	1
psg21	1				12	2	1	22	0	13	10	1	18	79	45	1.2	5
psg22	1	0	25	5	1	2	4	3	1	4	11	3	26	85	45	7.6	50
psg10	1	10	75	14	5	21	11	20	0	9	33	9	59	266	46	9	53
psg14	1	1	46	5	12	4	0	9	0	5	19	3	17	121	46	0.4	4
psg26	1	2	40	4	11	13	0				23	0	68	161	47.1	3.4	29
psg4	3				22	17	0							39	47.4	1.3	10
psg9	1	0	8	2	7	1	0	2	0	7	10	1	18	56	48	3.8	37
psg20	1	1	49	14	4	1	2	3	0	6	15	7	47	149	49	4.2	39
psg6	2				15	17	3							35	51.8	1.5	2
psg7[a]	3							9	23	1				33	62	4	5
psg25	1	5	19	17	1	16	16	0	5	1	19	5	28	132	66.2	3.3	2
psg29	1	0	20	3	1	16	34	2	0	5	17	0	3	101	70	6.2	9
psg11	1				0	18	23	0	15	4	24	1	0	85	73	2.7	2
psg24	1	0	14	7	0	18	7	2	8	30	13	14	0	113	79	1.1	1
psg13	1	5	8	12	2	1	13	0	8	7	4	4	0	64	82	2.3	5
psg18	1				8	13	27	1	4	42	4	22	0	121	88	1.2	4
psg17	1				3	11	37	1	8	33	1	3	1	98	100	3.2	0
psg12	1				3	1	16	2	8	37	1	28	19	115	102	1.9	7

This table displays the raw scoring data from crosses with four pairs of dominant markers. Each row represents the mapped PSG complementation groups. The third column from the right, represents the observed genetic map positions calculated using the formula in Figure 2B. The last two columns reflect the reliability of the mapping process and were calculated as described for *psg24* in Figure 2C. The raw data for *psg24* is also shown in Figure 2A. Mutants were sorted by cytological location.

[a] recombination results reported Wang *et al.* 2008

Table S3 Reliable mutant directionality even when *Gl,Sb* marker pair is not used.

Mutant	*R,D*	*Gl,+*	*+,Sb*	+,+	*Sb,H*	*H,Pr*
psg19	→	10	7	2	←	←
psg3	→	14	3	0		
psg27	→	6	1	0	←	←
psg21		12	2	1	←	←
psg22	→	1	2	4	←	←
psg10	→	5	21	11	←	←
psg14	→	12	4	0	←	←
psg15	→	11	13	0		←
psg9	→	7	1	0	←	←
psg20	→	4	1	2	←	←

Direction of recombination "splits" in the *R,D, Sb,H,* and *H,Pr* pairs accurately locate mutations to the *Gl,Sb* interval.
