# Supplementary material for: Rapid Recombination Mapping for High-Throughput Genetic Screens in Drosophila
Source: G3 (Bethesda). 2013 Oct 29;3(12):2313–9. doi: 10.1534/g3.113.008615 (PMC3852393; doi:10.1534/g3.113.008615)

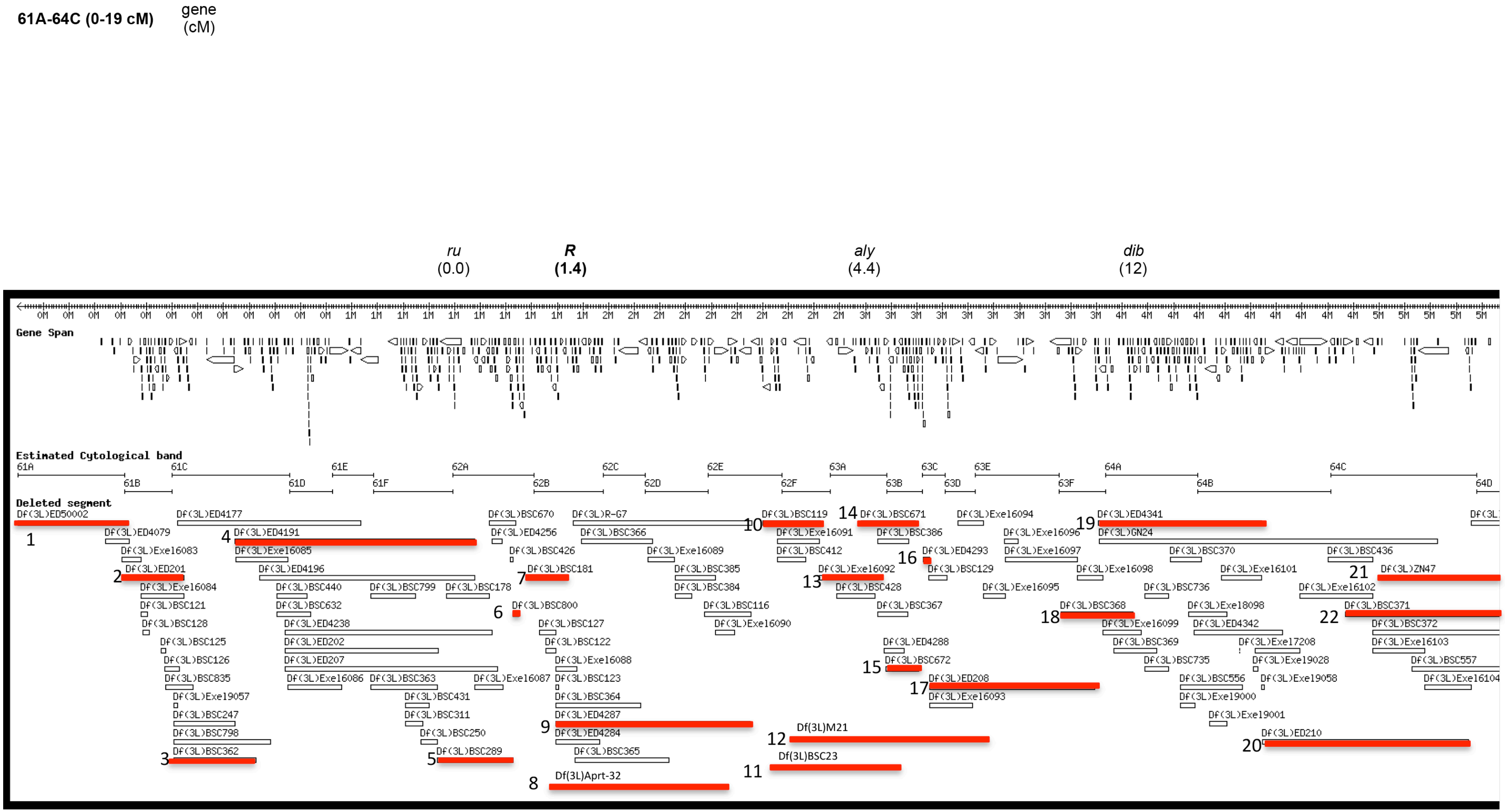

File S1. Modified map of the third chromosome showing BDSC deficiencies and the genetic map positions of reference genes. The map shows all deficiencies spanning the third chromosome (the 179 DK3 stocks are highlighted in red). Experimentally-derived genetic map positions of 42 genes (from Lindsley and Zimm 1992) are noted above the map to facilitate conversions between genetic and cytological map positions. In order to simplify printing, the map is split into 9 consecutive pages (with a small amount of overlapping information). Modified from a chromosome 3 deficiency map available on the Bloomington Drosophila Stock Center website (http://flystocks.bio.indiana.edu/pdfs/DfMap3.pdf).

64D-67F (19-34 cM) gene (cM)

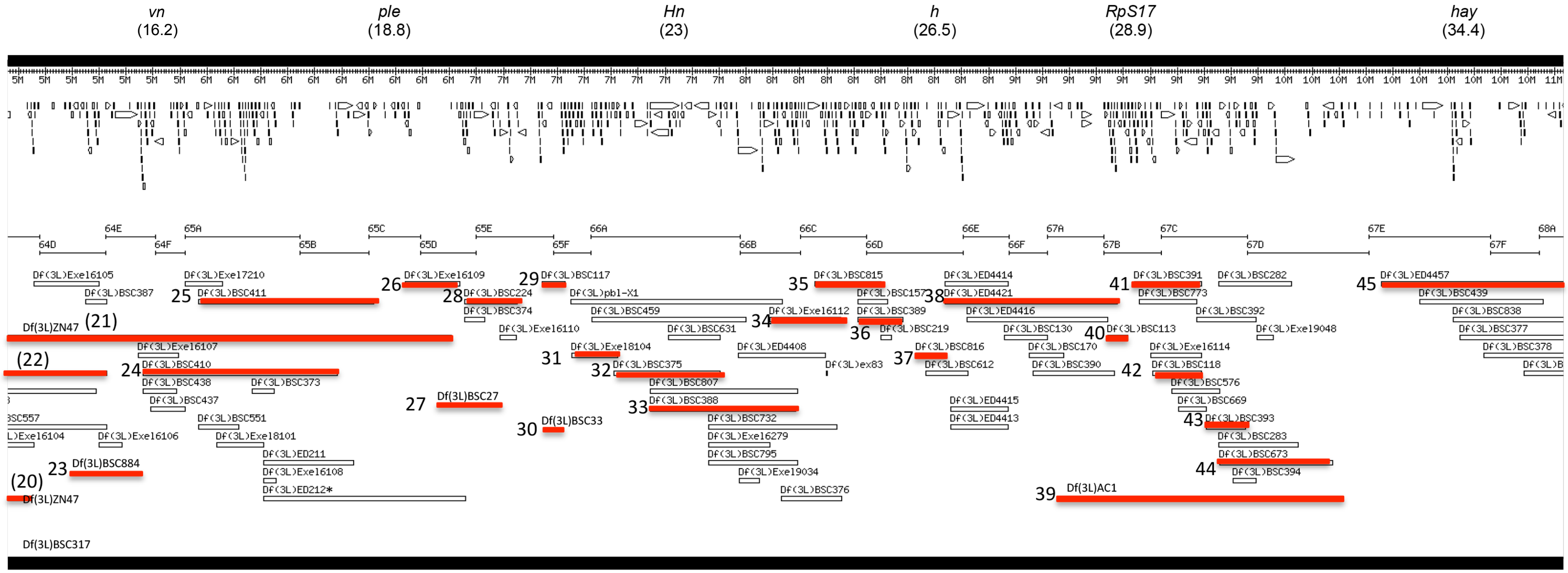

68A-73A (35-44 cM) gene (cM)

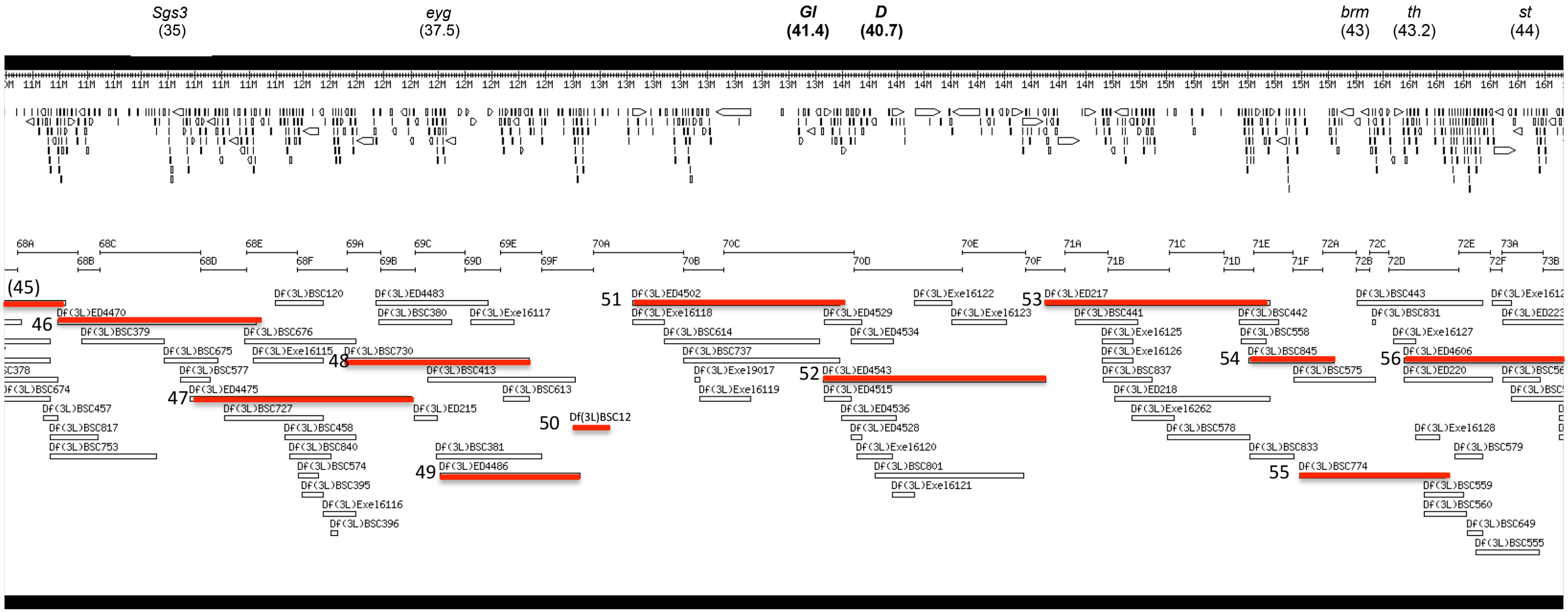

73B-79D (44-47 cM) gene (cM)

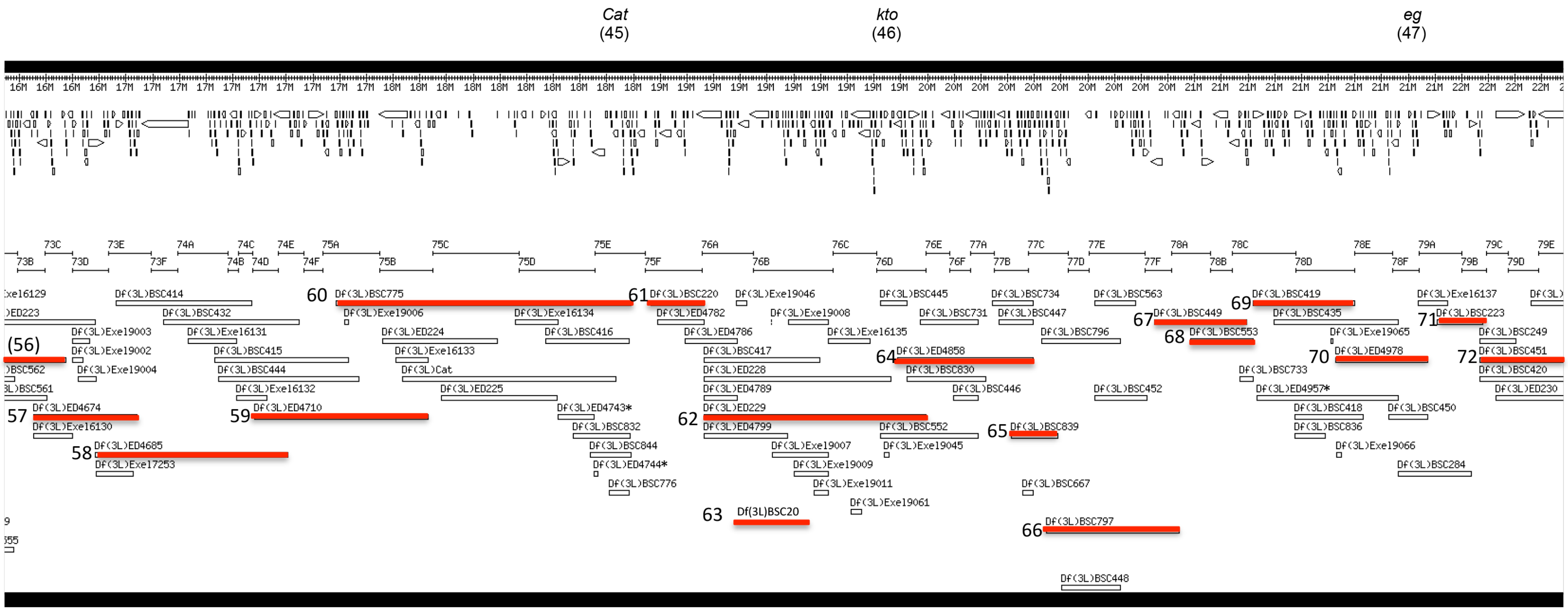

79C-85C (47-48 cM) gene (cM)

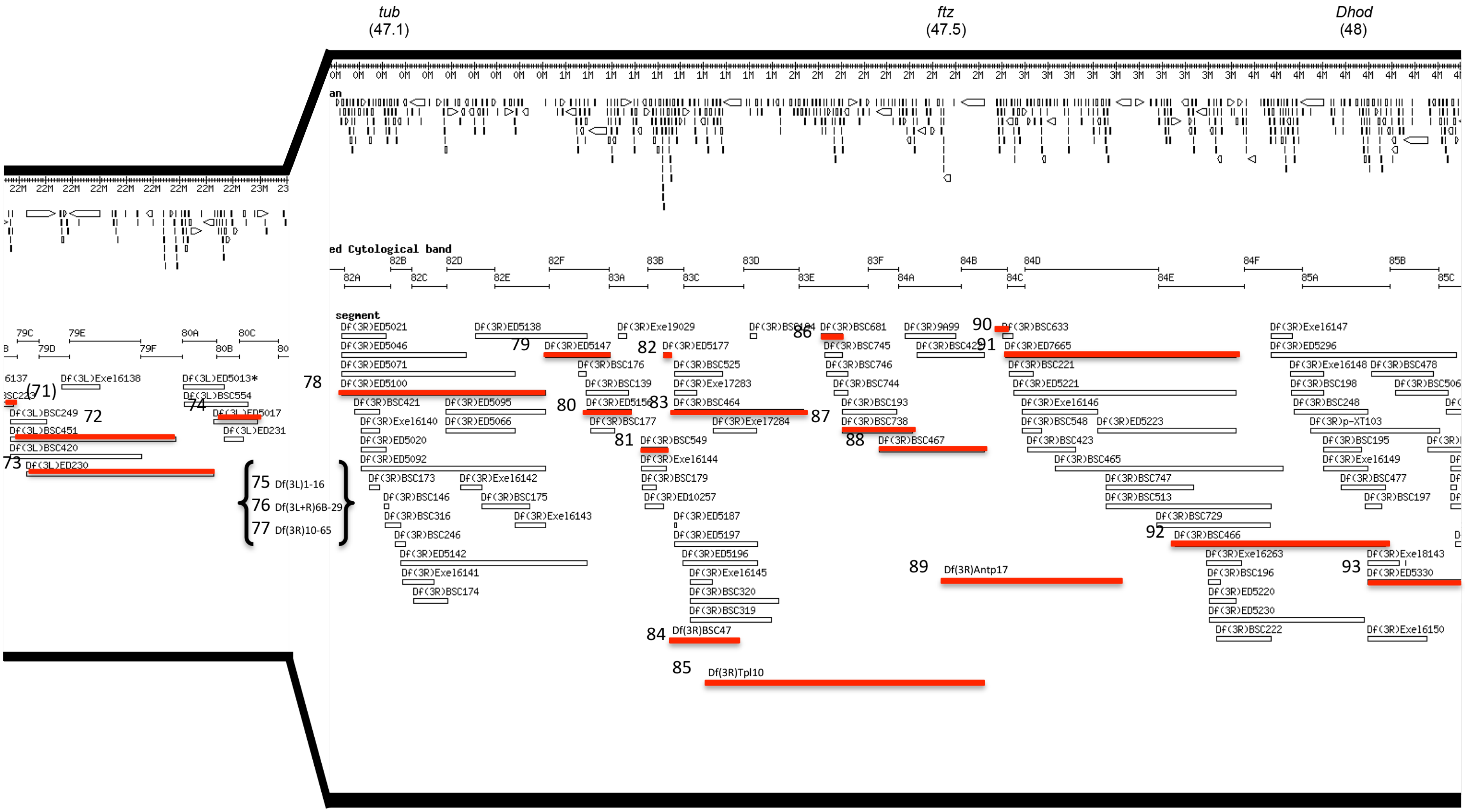

85C-88E (48.5-54 cM) gene (cM)

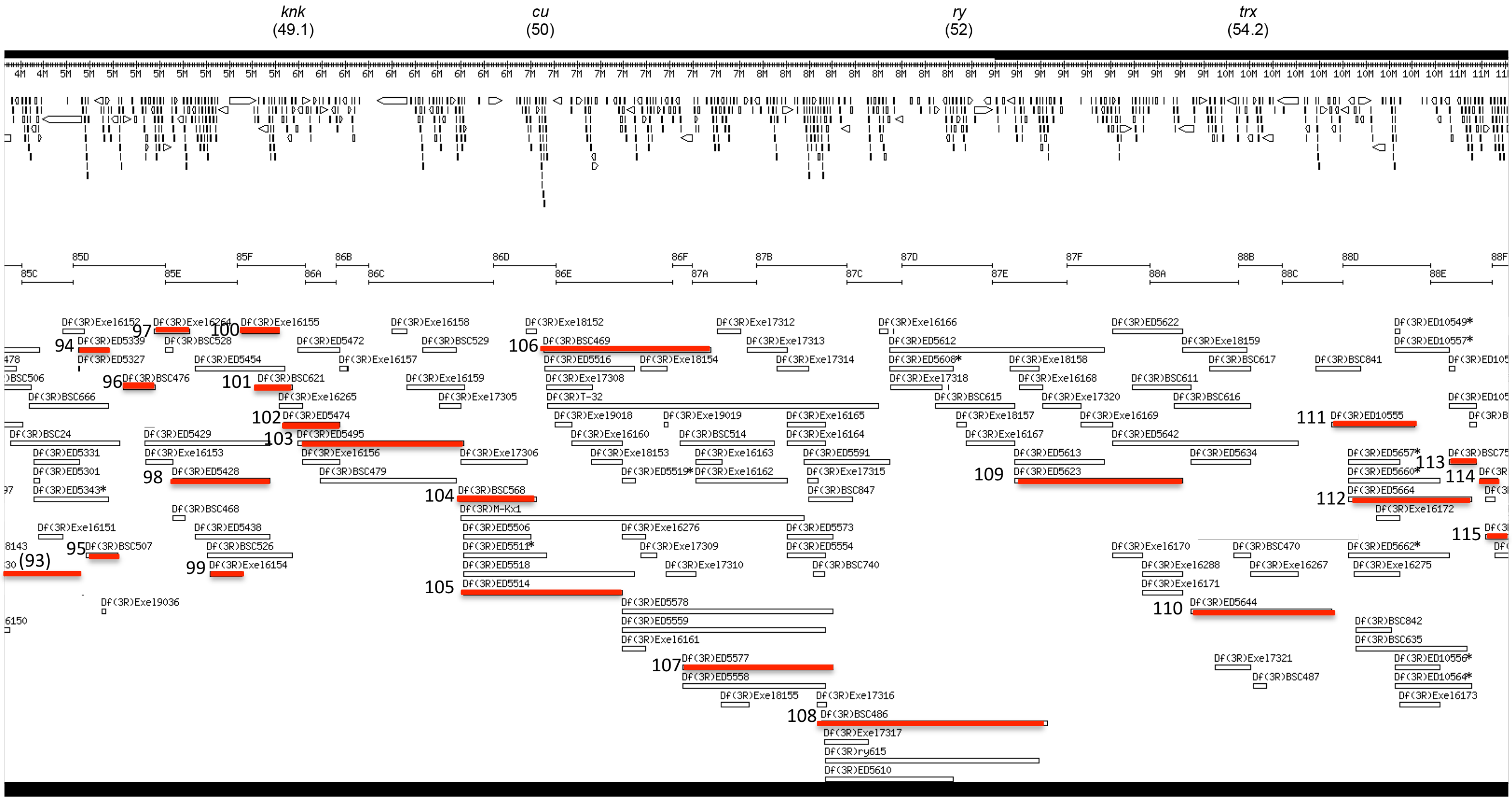

88F-93E (57-72 cM) gene (cM)

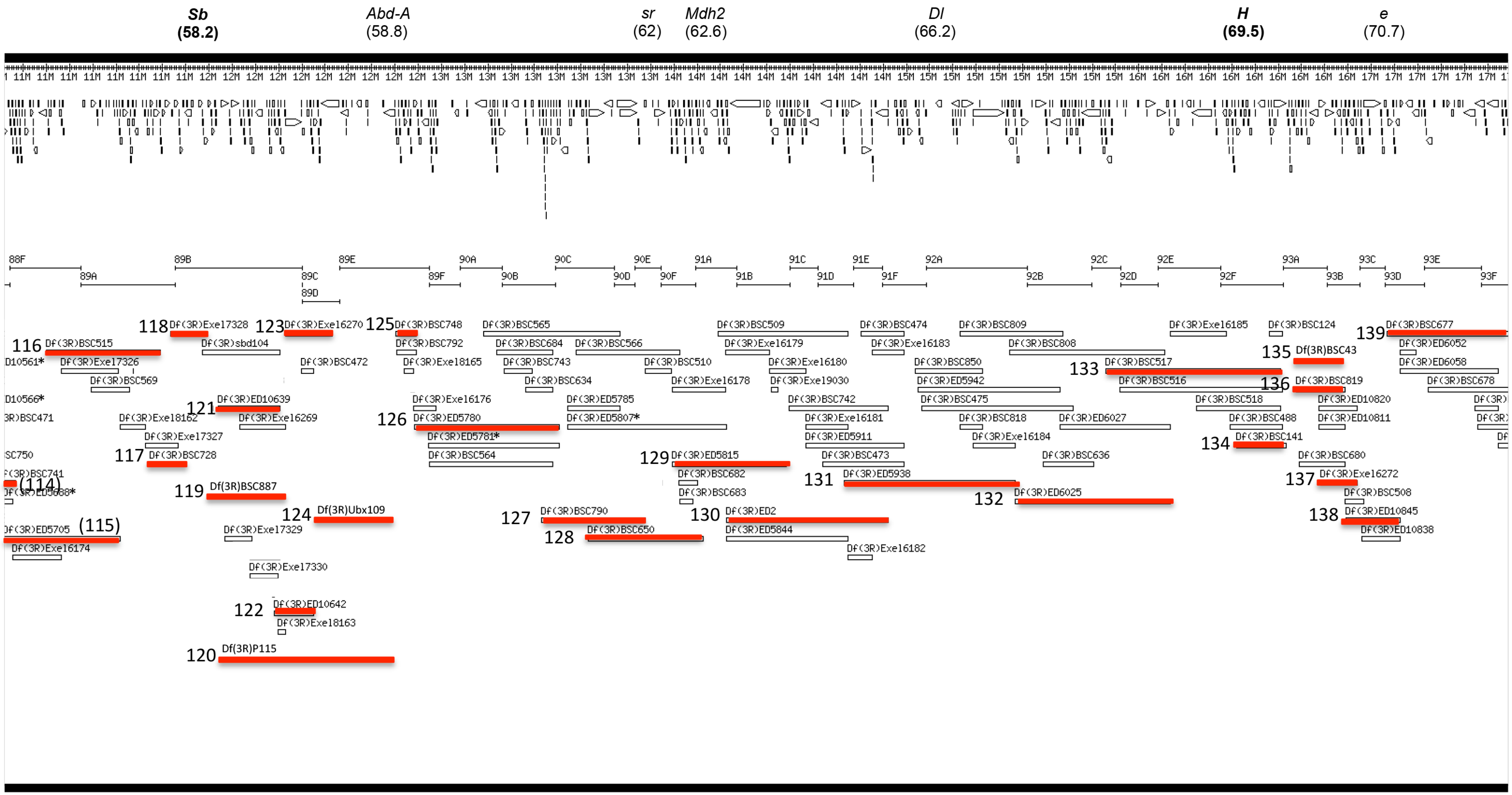

93D-97F (73-98 cM) gene (cM)

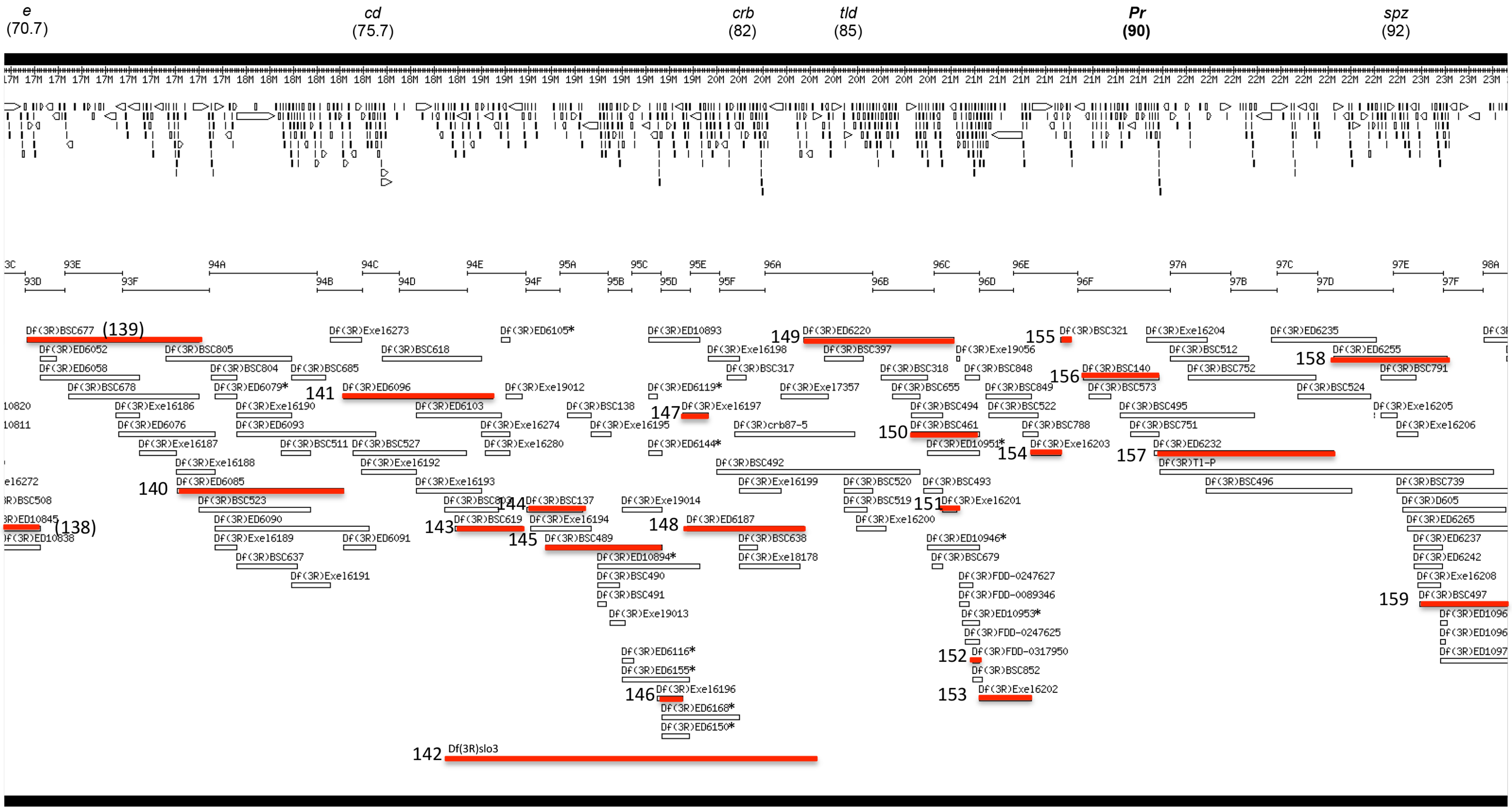

97A-110E (90-110 cM) gene (cM)

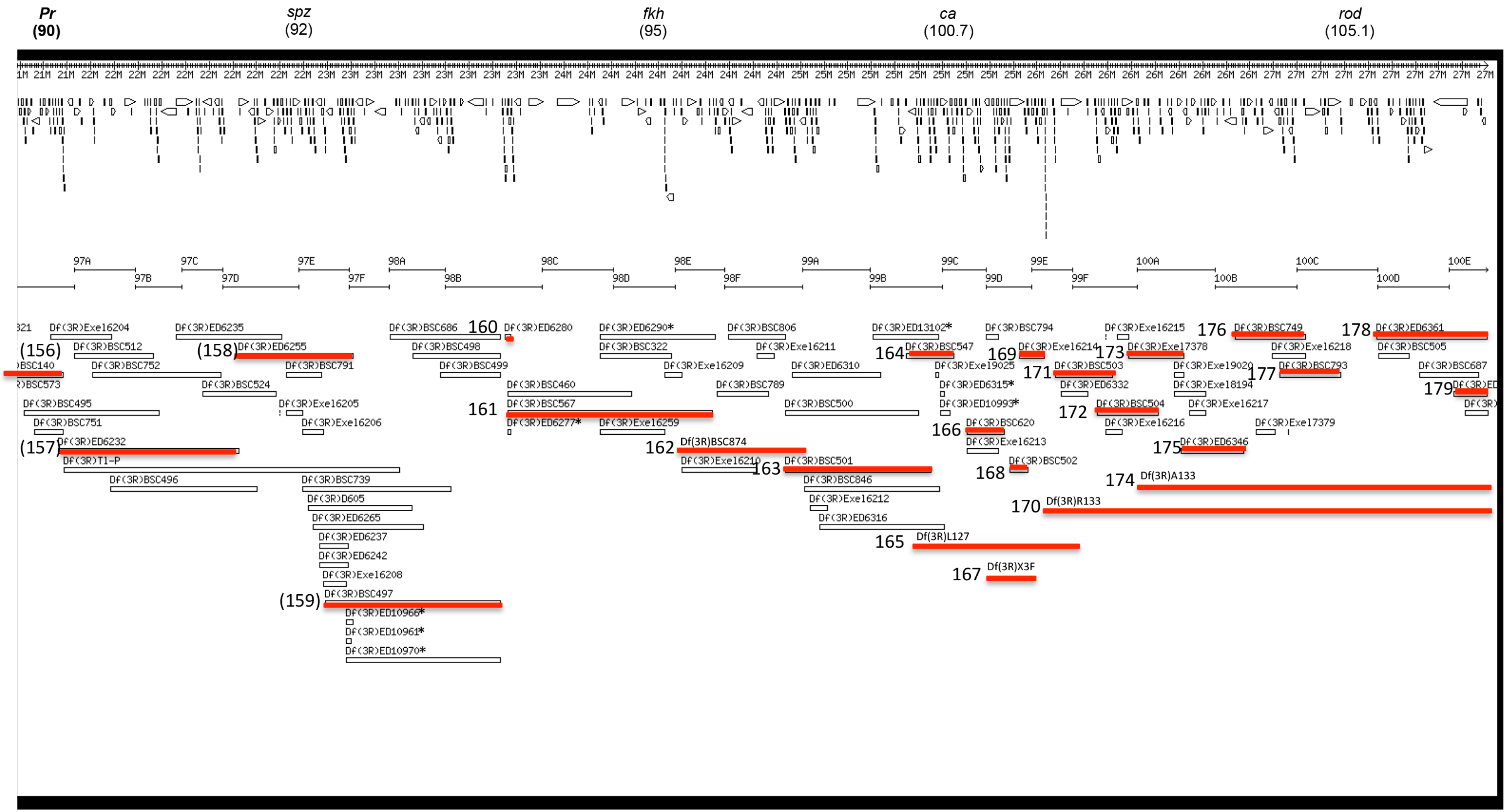

Supplement: Supporting Information [file supp_g3.113.008615_FileS1.pdf]
