# Supplementary material for: Rapid Recombination Mapping for High-Throughput Genetic Screens in Drosophila
Source: G3 (Bethesda). 2013 Oct 29;3(12):2313–9. doi: 10.1534/g3.113.008615 (PMC3852393; doi:10.1534/g3.113.008615)
Supplement: Supporting Information [file supp_g3.113.008615_TableS1.pdf]

Table S1   Useful dominant markers for mapping on the second chromosome

genotype	name	cM	cytology
S[1]	*Star, Asteroid*	2-1.3	21E4
wg[Sp-1]	*Sternopleural*	2-22	27F1
J[1]	*Jammed*	2-41	31E
amos[Tft]	*Tufted*	2-53.6	36F6
Bl[1]	*Bristle*	2-54.8	38B5
L[rm] or L[2]	*Lobe*	2-72	51A4
nw[D] or nw[B]	*narrow*	2-79.6	54A1
Bc[1]	*Black cells*	2-80.6	54F6
Pu[2]	*Punch*	2-97	57C7
Frd[1]	*Freckled*	2-102.4	59C1-4
Pin[1]	*Pin*	2-107.3	60C6-D1

Most available as stocks in various multiply-marked combinations from BDSC
