# Supplementary material for: Rapid Recombination Mapping for High-Throughput Genetic Screens in Drosophila
Source: G3 (Bethesda). 2013 Oct 29;3(12):2313–9. doi: 10.1534/g3.113.008615 (PMC3852393; doi:10.1534/g3.113.008615)
Supplement: Supporting Information [file supp_g3.113.008615_TableS2.pdf]

Table S2 Viable F2 recombinant progeny data generated by recombination analysis using pairs of dominant markers.

		Recombination mapping F2 progeny															
mutation	alleles	*R,D*			*Gl,Sb*			*Sb,H*			*H,Pr*			total scored	cM	cM away	Dfs away
		R,+	*+,D*	*+,+*	*Gl,+*	*+,Sb*	*+,+*	*Sb,+*	*+,H*	*+,+*	*H,+*	*+,Pr*	*+,+*				
psg8	2	3	1	4	9	4	27				11	0	15	74	0	0	2
psg28	1	25	3	7	4	1	31	21	12	83	13	4	51	255	0	0	2
psg2[a]	2	28	42	1										71	15	10	5
psg5[a]	2	20	91	1										112	25	9	9
psg15	1	9	19	0	7	2	19				15	0	15	86	25.2	2.9	6
psg23	1	7	9	0	7	0	17	2	0	17	10	3	25	97	28	4.5	4
psg16	1	0	26	0	3	1	0				2	0	4	36	35	5.7	6
psg19	1	0	11	2	10	7	2	13	4	59	27	0	26	161	43.6	4.7	39
psg3	2	0	124	9	14	3	0							150	43.6	0.8	1
psg27	1	0	5	1	6	1	0	14	1	6	14	3	13	64	43.6	0.2	1
psg21	1				12	2	1	22	0	13	10	1	18	79	45	1.2	5
psg22	1	0	25	5	1	2	4	3	1	4	11	3	26	85	45	7.6	50
psg10	1	10	75	14	5	21	11	20	0	9	33	9	59	266	46	9	53
psg14	1	1	46	5	12	4	0	9	0	5	19	3	17	121	46	0.4	4
psg26	1	2	40	4	11	13	0				23	0	68	161	47.1	3.4	29
psg4	3				22	17	0							39	47.4	1.3	10
psg9	1	0	8	2	7	1	0	2	0	7	10	1	18	56	48	3.8	37
psg20	1	1	49	14	4	1	2	3	0	6	15	7	47	149	49	4.2	39
psg6	2				15	17	3							35	51.8	1.5	2
psg7[a]	3							9	23	1				33	62	4	5
psg25	1	5	19	17	1	16	16	0	5	1	19	5	28	132	66.2	3.3	2
psg29	1	0	20	3	1	16	34	2	0	5	17	0	3	101	70	6.2	9
psg11	1				0	18	23	0	15	4	24	1	0	85	73	2.7	2
psg24	1	0	14	7	0	18	7	2	8	30	13	14	0	113	79	1.1	1
psg13	1	5	8	12	2	1	13	0	8	7	4	4	0	64	82	2.3	5
psg18	1				8	13	27	1	4	42	4	22	0	121	88	1.2	4
psg17	1				3	11	37	1	8	33	1	3	1	98	100	3.2	0
psg12	1				3	1	16	2	8	37	1	28	19	115	102	1.9	7

This table displays the raw scoring data from crosses with four pairs of dominant markers. Each row represents the mapped PSG complementation groups. The third column from the right, represents the observed genetic map positions calculated using the formula in Figure 2B. The last two columns reflect the reliability of the mapping process and were calculated as described for *psg24* in Figure 2C. The raw data for *psg24* is also shown in Figure 2A. Mutants were sorted by cytological location.

[a] recombination results reported Wang *et al.* 2008
