# Supplementary material for: Rapid Recombination Mapping for High-Throughput Genetic Screens in Drosophila
Source: G3 (Bethesda). 2013 Oct 29;3(12):2313–9. doi: 10.1534/g3.113.008615 (PMC3852393; doi:10.1534/g3.113.008615)
Supplement: Supporting Information [file supp_g3.113.008615_TableS3.pdf]

Table S3 Reliable mutant directionality even when *Gl,Sb* marker pair is not used.

Mutant	*R,D*	*Gl,+*	*+,Sb*	+,+	*Sb,H*	*H,Pr*
psg19	→	10	7	2	←	←
psg3	→	14	3	0		
psg27	→	6	1	0	←	←
psg21		12	2	1	←	←
psg22	→	1	2	4	←	←
psg10	→	5	21	11	←	←
psg14	→	12	4	0	←	←
psg15	→	11	13	0		←
psg9	→	7	1	0	←	←
psg20	→	4	1	2	←	←

Direction of recombination “splits” in the *R,D, Sb,H,* and *H,Pr* pairs accurately locate mutations to the *Gl,Sb* interval.
